# The Relationship between Fear of Childbirth and Women's Knowledge about Painless Childbirth

**DOI:** 10.1155/2014/274303

**Published:** 2014-11-12

**Authors:** Mehmet Aksoy, Ayse Nur Aksoy, Aysenur Dostbil, Mine Gursac Celik, Ilker Ince

**Affiliations:** ^1^Department of Anaesthesiology and Reanimation, Faculty of Medicine, Ataturk University, 25240 Erzurum, Turkey; ^2^Department of Obstetrics and Gynaecology, Nenehatun Hospital, 25070 Erzurum, Turkey

## Abstract

This study investigated the association between fear of childbirth (FOC) and women's knowledge about painless childbirth methods. The study was performed on 900 multiparous women within the last month of pregnancy. Data was obtained through a questionnaire including the Wijma Delivery Expectancy/Experience Questionnaire (W-DEQ) Turkish form A. FOC was defined as W-DEQ sum score ≥85. Women were questioned about their knowledge about painless childbirth and the most important source of this knowledge. Group 1 consists of participants with knowledge about painless childbirth. Group 2 consists of participants without knowledge about painless childbirth. Five hundred and twenty-four women (58.2%) had knowledge while 376 women (41.7%) had no knowledge about painless childbirth. Mean W-DEQ scores in group 1 (68.46 ± 12.53) were found to be lower than group 2 (71.35 ± 12.28) (*P* = 0.001). FOC was associated with increased maternal request for elective caesarean section (OR 4.22, 95% CI 2.91–6.11). Better informed pregnant women about painless childbirth methods may reduce the number of women with FOC and the rate of preferred elective caesarean section.

## 1. Introduction

Fear of childbirth (FOC) is a serious problem for women, since it leads to avoidance of pregnancy, maternal and fetal stress, and an increase in maternal requests for cesarean section [1]. Some studies have been conducted to investigate the causes of this fear [2, 3]. Størksen et al. [2] found a strong association between a previous subjectively negative birth experience and FOC. Sluijs et al. [3] showed that fear levels were higher in nulliparous women compared to multiparous women.

Normal vaginal birth for women is a painful event due to uterine contractions, recurrent vaginal examinations, and vaginal lacerations. For most women, childbirth is associated with very severe pain. The perceived pain during labour causes generalised neuroendocrinal stress response including increased oxygen consumption, hyperventilation, increased cardiac output, impaired uterine contractility, metabolic acidemia, and increased maternal-fetal mortality and morbidity [4]. So, many labor pain management strategies named painless childbirth methods have been developed and widely used in recent years [5, 6]. Painless childbirth methods include pharmacologic (such as regional anesthesia, paracervical block, pudendal block, and systemic analgesia) and nonpharmacologic methods (such as psychoprophylactic method, hypnosis, and acupuncture).

Women may change their preferred delivery method due to fear of labour pain and this is one of the most important reasons of the increase in the rate of elective cesarean section in recent years [7, 8]. Studies have shown that women with FOC require more use of pain relieving methods in labour compared to women without FOC [7].

We hypothesized that if women have knowledge about painless childbirth, lower rate of FOC may be in these patients compared to those without knowledge about painless childbirth. Thus, aim of this study was to investigate the association between FOC and women's knowledge about painless childbirth in multiparous women with uncomplicated pregnancy.

## 2. Materials and Methods

This study was conducted at Nenehatun Hospital, Erzurum, Turkey, a metropolitan teaching hospital performing approximately 6000 deliveries/year. Nine hundred women in their final month of pregnancy reviewed at the hospital for antenatal care and decisions regarding delivery type between January 1, 2012 and May 1, 2013 were selected for this study. The protocol of the study was approved by the Ethics Committee of our institute (the protocol number: 12) and informed consent was obtained from all of the participants. Only multiparous patients with positive experiences were included in this study to minimize other factors causing FOC such as nulliparity and previous experience of a traumatic birth. Initially, obstetric examination was performed to detect whether there was a maternal or fetal problem. Women's demographic-obstetric information was recorded and patients were questioned for the determination of whether they have inclusion criteria for this current study using a semistructured interview technique (Appendix A). Women with complicated pregnancies (e.g., preeclampsia, fetal malformation, gestational diabetes mellitus, and placenta previa), chronic illnesses (e.g., hypertension and diabetes mellitus), and multiple pregnancies were excluded from the study. Patients were questioned about their birth experience and the answers were analysed [9]. Women who had had a negative birth experience (numeric rating scale score (NRS) ≥ 9) were excluded. Patients who delivered vaginally with regional anaesthesia in previous pregnancy and who had previous caesarean delivery were also excluded.

A questionnaire including the Wijma Delivery Expectancy/Experience Questionnaire (W-DEQ) form A was completed by the patients who have inclusion criteria for this study. W-DEQ form version A, which is a prepartum version of the scale, has a 33-item assessing FOC level according to women's cognitive appraisal and expectancies about delivery (items like “How do you think you will feel in general during the labour and delivery?” Extremely weak-not at all weak, extreme panic-not at all panicked, extreme trust-no trust at all). It was shown that W-DEQ form version A had good internal consistency with a Cronbach's *α* coefficient of 0.93 [10]. Each item has six scale points ranging from 0 to 5 and total scores ranged from 0 to 165. FOC is defined as W-DEQ sum score ≥85, and severe FOC is defined as W-DEQ ≥100 [10]. Turkish form of W-DEQ version A was found to be reliable and valid (internal consistency coefficient for the WDEQ was 0.89) in a sample of 660 Turkish pregnant women by Korukcu et al. [11] and was used to detect FOC in this study. Participants' responses to questions were recorded.

Following completed W-DEQ form A, participants were questioned about whether they had knowledge about painless childbirth, the method of painless childbirth which they had maximum knowledge of, the most important source of this knowledge, and their preferred delivery method for their current pregnancy (Appendix B). Participants were also asked about whether they preferred painless childbirth for current pregnancy, after informing them about painless childbirth methods (Appendix B). Individual informing was performed by an obstetrician to evaluate whether positive information about painless childbirth methods has an effect on caesarean delivery preference. Applicable pain relief methods and possible interventions and alternatives for pain relief during labor were explained without technical details for about one hour verbally. Questions of the patients with suspicions about the painless childbirth methods (such as the stroke risk due to the procedure) were answered.

A power analysis for this study was calculated based on the work of Fenwick et al. [12] using Russ Lenth's Power and sample size calculation application [13]. It was calculated that 435 women were required to give 80% power (alpha 5%) to detect a 5% difference in FOC between groups 1 and 2, if FOC was 10% in group 2 and 5% in group 1.

SPSS software 12.0 (SPSS Inc., Chicago, IL, USA) was used for the statistical analysis. The Kolmogorov-Smirnov test was used to determine whether data had normal distribution. The unpaired *t* test was used for analysis of participants for differences in demographic and obstetric characteristics. Participants were divided two groups according to their knowledge about painless childbirth:* group 1*: participants with knowledge about painless childbirth and* group 2*: participants who do not have knowledge about painless childbirth. Mean W-DEQ scores of the two groups were compared using the unpaired *t* test. The Pearson Chi-square test was used for comparison of women with FOC according to whether they desired painless childbirth. The Chi-square test was used to compare the preferred delivery methods of patients with or without FOC in two groups. The data were calculated as mean ± standard deviation and odds ratios (OR) with 95% confidence interval (CI). *P* < 0.05 was considered as significant.

## 3. Results

**Figure 1:**
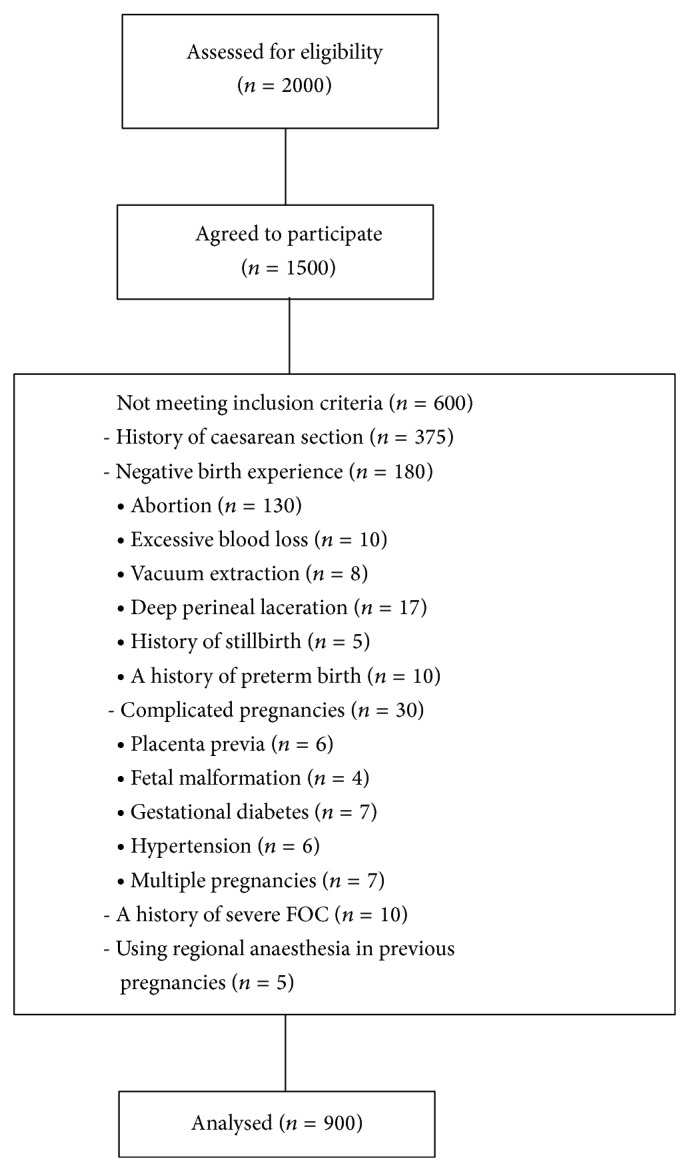
Flow of participants into the study.

Flow of participants into the study is shown in Figure 1. Of 2000 eligible women, 1500 agreed to participate and 600 had exclusion criteria, leaving a final study sample of 900 included women.

The women's clinic characteristics and mean W-DEQ scores are presented in Table 1. Five hundred and twenty-four women (58.2%) were knowledgeable (group 1), while 376 women (41.7%) had little knowledge about painless childbirth (group 2). Mean W-DEQ scores of participants in both groups were 69.6 ± 12.4 (range, 5–99). Mean W-DEQ scores in group 1 were found to be lower (68.46 ± 12.53) than in group 2 (71.35 ± 12.28) (*P* = 0.001). The percentage of patients diagnosed with FOC in group 2 (*n* = 76, 20.2%) was higher than in group 1 (*n* = 69, 13.2%) (*P* = 0.005, OR 1.67, 95% CI 1.1–2.3, Table 1). There was no severe FOC (W-DEQ ≥ 100) in both of groups.

Regarding the painless childbirth method about which participants in group 1 had most knowledge, 300 (57.2%) patients knew that painless childbirth was provided by inserting a needle in the lower back, and 150 (28.6%) patients knew that it was provided by intramuscular or intravenous drug administration during labour pain (Table 2). Most of the patients (272, 51.9%) said that they have received most information from their friends (Table 2). There were a total of 145 patients with FOC in the two groups. The percentage of patients with FOC was similar amongst university graduates (98/159, 61.6%) and those without a university degree (427/741, 57.5%) (*P* > 0.05).

**Figure 2:**
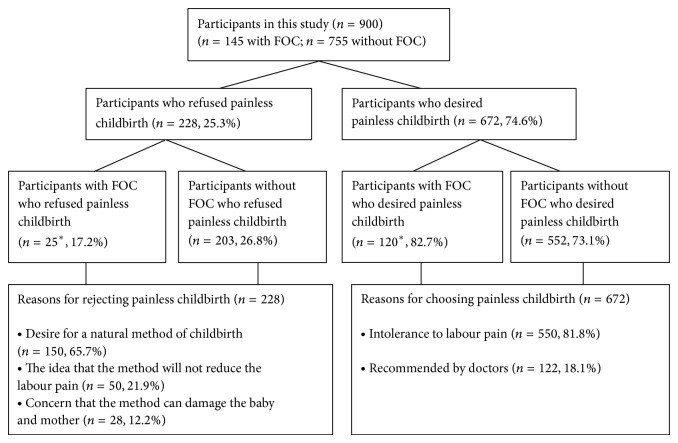
Distribution of participants according to whether they preferred painless childbirth after being informed about painless childbirth. FOC: fear of childbirth, ^*^ *P* < 0.001 compared to participants without FOC.

The relationship between FOC and maternal preference for delivery method is shown in Table 3. The odds ratio of knowledge about painless childbirth was 5.72 (95% CI 4.1–7.9) for preferring delivery methods. The analysis of our data shows that request for caesarean section in 66.5% of women resulted from fear of labour pain (Table 3). There were a total of 145 women with FOC, 78 (53.7%) of them desired elective caesarean section. FOC was associated with preference for caesarean section (OR 4.22, 95% CI 2.91–6.11). After they were informed positively about the methods of painless childbirth, 53 pregnant women changed their mind and chose vaginal delivery with painless childbirth methods. Thus, the proportion of women requesting caesarean section dropped from 53.7% to 17.2% in women with FOC and FOC was associated with preferring painless childbirth (OR 13.05, 95% CI 8.24–20.68). Two hundred and twenty-eight of all patients (25%) refused the methods of painless childbirth for their current pregnancy due to various reasons (Figure 2).

## 4. Discussion

In this study, we researched the relationship between FOC and knowledge about painless childbirth in multiparous women with a positive birth experience. Nine hundred women answered the questionnaire, 524 of them were knowledgeable and 376 of them were unknowledgeable about painless childbirth. Mean W-DEQ scores and the percentage of patients with FOC were significantly higher in women with little knowledge about painless childbirth than in women who have knowledge about it. There were a total of 145 patients with FOC in the study group (16.1%). Severe FOC was not reported in our study. Whether or not they experienced FOC, most of the patients who were knowledgeable about painless childbirth chose vaginal birth. After positively informing them about the painless childbirth, almost all of the women with FOC desired painless childbirth.

Although numerous studies have been conducted for decades about FOC [1–3], the exact causes and treatment of FOC have not been found as yet. Størksen et al. [2] found strong association between previous subjectively negative birth experience and FOC in the subsequent pregnancy. Nilsson et al. [9] also showed an association between FOC and negative birth experiences. Additionally, it has been indicated that nulliparous women had higher mean W-DEQ scores than parous women [14]. To minimize the factors that caused FOC, multiparous patients with no negative birth experiences only enrolled in this current study researching the relationship between FOC and knowledge about painless childbirth.

Turkish form of W-DEQ version A was used in this current study to measure the degree of fear of childbirth in participants. Previously, it was showed that this form had satisfactory internal consistency and Cronbach's alpha (0.89) for the Turkish version of the W-DEQ. These results were similar to the results of the developers of the scale in addition to results of the British version [10, 15]. Mean W-DEQ scores of participants in this current study were 69.6 ± 12.4 (range, 5–99). Similar W-DEQ scores were reported by Fenwick et al. [12] and Rouhe et al. [16] (57.81 ± 19.66 (range 6–115) and 68.3 ± 21.1 (range 9–160), resp.). But the score range in their study was different from the score range in this current study. As the reason for this difference, it may be said that the women with negative birth experiences were excluded from this current study population. So, there were no women with W-DEQ scores ≥ 100 in this current study.

Pregnancy and the delivery processes may be intolerable for women with FOC. In a study [17] examining the intensity and type of childbirth fears, fear for the child's health and fear of pain were found to be the most frequent fears. The researchers reported that most women with FOC chose to have a caesarean section rather than vaginal delivery because of fear of labour pain [17]. Similar to these results, caesarean section was preferred in a higher rate than vaginal delivery by women with FOC compared to those without FOC in our study.

In our hospital, painless childbirth methods have been applied to the patients who wish painless childbirth and also elective caesarean section for maternal request has not been performed. Unfortunately, a large proportion of women do not have knowledge about the methods of painless childbirth. The incidence of awareness and acceptance of labour analgesia have been reported as 9.5 and 23% in the Indian population [18], 27 and 57.6% in the Nigerian population [19], and 98 and 80% in the Australian population [20]. However, the percentage of women who have knowledge about painless childbirth was found to be as 58.2% in our study. But some of these patients had suspicions and fears about the pain relief methods. After positive informing about painless childbirth methods of all patients, acceptance of painless childbirth was 74.6% in all women, 83% in women with FOC, and 73.1% in women without FOC in this current study. The most important reason for refusal of painless childbirth among patients was the request to have a natural birth. Also the percentage of women defined as FOC was higher in women who were unknowledgeable about painless childbirth than in women who were knowledgeable about it in our study.

In our study, with respect to the source of their knowledge about painless childbirth, more women received knowledge from their friends, similar to Naithani et al.'s [18] study. However, the anaesthetist or obstetrician was reported as a source of information in the Australian population [20]. The reason for this difference may be that most of our study population consisted of people who had not graduated from university.

One target for reducing rates of elective caesarean section is to reduce rates of caesarean section for maternal request, which have been increasing [21, 22]. A relationship has previously been found between FOC and maternal request for elective caesarean section [21]. Sydsjö et al. [23] showed that secondary FOC prolongs the time to subsequent delivery and the active phase of labour itself and increases the risk for caesarean section. Furthermore, we reported that women (FOC or not) with knowledge about painless childbirth requested a lower choosing rate of caesarean section compared to women without knowledge about it. We also reported a lower rate of caesarean section request after positively informing women with FOC about painless childbirth.

The limitation of this study is that what the mode of birth ended up being is not actually known.

## 5. Conclusions

Fear of labour pain was found to be the major cause of Turkish women requesting elective caesarean section. Turkish women's knowledge about painless childbirth methods is insufficient. Better informing pregnant women about painless childbirth methods may reduce the number of women with FOC, the severity of fear in women, and the rate of choosing elective caesarean section due to fear of labour pain. Also, it may increase the use of painless childbirth methods by women. Future studies including postpartum data need to detect the association between fear of childbirth and women's knowledge about painless childbirth.

## Figures and Tables

**Table 1:** Women's demographic and obstetric characteristics and mean W-DEQ scores.

Characteristic	Group 1 (*n* = 524, 58.2%)	Group 2 (*n* = 376, 41.7%)	*P* value
Age (years)	29.52 ± 4.31	29.96 ± 4.37	>0.05
Parity	2.20 ± 0.54	2.22 ± 0.51	>0.05
Gestational week	35.87 ± 0.85	35.91 ± 0.85	>0.05
BMI (kg/m^2^)	29.18 ± 3.30	28.83 ± 2.94	>0.05
Mean W-DEQ scores	68.46 ± 12.53	71.35 ± 12.28	=0.001
W-DEQ scores (min, max)	5, 95	5, 99	
Patients with FOC (*n*, %)	69, 13.2%	76, 20.2%	=0.005
University graduates (*n*, %)	94, 17.9%	65, 17.2%	>0.05

Group 1: participants with knowledge about painless childbirth, group 2: participants who do not have knowledge about painless childbirth.

**Table 2:** Methods of painless childbirth about which participants had knowledge.

Method of painless childbirth	Number of participants that have knowledge
Provided by inserting a needle in the lower back	300, 57.2%

Provided by intramuscular or intravenous drug administration	150, 28.6%

Other (e.g., acupuncture, deep breathing exercise)	74, 14.1%

Source of knowledge about painless childbirth *n*, %	My friends: 272 (51.9%), Television: 29 (5.53%), Internet: 87 (16.6%), Doctor: 101 (19.2%), Nurse: 35 (6.67%).

**Table 3:** Delivery preference of participants and reasons for requesting elective caesarean section.

	Patients preferring caesarean section for their current pregnancy	Patients preferring vaginal delivery for their current pregnancy
	*n*, %	*n*, %
The patients with FOC in group 1 (*n* = 69)	8^*^, 11.5	61^*^, 88.4

The patients with FOC in group 2 (*n* = 76)	70, 92	6, 7.8

The patients without FOC in group 1 (*n* = 455)	55^**^, 12	400^*^, 87.9

The patients without FOC in group 2 (*n* = 300)	100, 33.3	200, 66.6

Causes for preferring caesarean section (*n* = 233)	(i) Fear of pain caused by uterine contractions (*n* = 155, 66.5%)	
	(ii) Control request delivery time (*n* = 50, 21.4%)	
	(iii) Fear of perineal tear (*n* = 19, 8.1%)	
	(iv) Request of tubal ligation (*n* = 9, 3.8%)	

Group 1: participants with knowledge about painless childbirth, group 2: participants who do not have knowledge about painless childbirth. ^*^ *P* < 0.0001, compared to the patients with FOC in group 2; ^**^ *P* < 0.001, compared to the patients without FOC in group 2.

## Appendix

### A. The First Questionnaire Form Used for Multiparous Patients in this Study Population

(A)
  (i) The first letters of first and last name:
  (ii) Phone number:
  (iii) Age:
  (iv) Length:
  (v) Weight:
  (vi) Parity:
  (vii) Gestational week:
  (viii) The level of education:
    (a) University graduate
    (b) Non-university graduate
(B)
  (i) Previous birth experiences.
    (a) A history of cesarean section:
    (b) A history of being diagnosed with severe fear of childbirth in previous pregnancies:
    (c) A history of negative birth experience:
      (1) abortion
      (2) excessive blood loss
      (3) vacuum extraction
      (4) deep perineal laceration
      (5) stillbirth
      (6) preterm birth
      (7) other:
  (ii) Complicated situations in the current pregnancy:
    (a) placenta previa
    (b) fetal malformation
    (c) gestational diabetes
    (d) hypertension
    (e) multiple pregnancy
    (f) other:
  (iii) A history of using regional anesthetic methods in previous births:
  (iv) Numeric rating scale (NSR) score: Please give a number for the overall experience of your birth from 0 (very good) to 10 (extremely bad):

^∗∗^If you have any of the conditions in B section or your NSR score is nine or more, please return the questionnaire form to interviewer. If you do not have any of the conditions in B section or your NSR score is eight or less, you may continue to respond the questions in the Wijma Delivery Expectancy/Experience Questionnaire form A.

### B. The Questionnaire Form Used after Completed Wijma Delivery Expectancy/Experience Questionnaire Form A for Multiparous Patients in this Study Population

(C)
  (i) Have you knowledge about painless childbirth?
    (a) Yes
    (b) No
  (ii) Which is the method of painless childbirth that you had maximum knowledge about it?
    (a) It is provided by inserting a needle in the lower back
    (b) It is provided by intramuscular or intravenous drug administration
    (c) Other (Acupuncture, deep breathing exercise, e.g.)
  (iii) What is the most important source of this knowledge for you?
    (a) My friends
    (b) Television
    (c) Internet
    (d) Doctor
    (e) Nurse
    (f) Other
  (iv) What is your preferred birth type for the current pregnancy?
    (a) Normal vaginal delivery
    (b) Elective caesarean section
  (v) Which is the most important reason in choosing caesarean section for you?
    (a) Fear of pain caused by uterine contractions
    (b) Control request delivery time
    (c) Fear of perineal tear
    (d) Request of tubal ligation
    (e) Other

Please contact your obstetrician, before answering the following questions. You will be informed about applicable pain relief methods and possible interventions and alternatives for pain relief during labor by your obstetrician for about one hour.

^∗∗^Please answer the following questions after informing by your obstetrician.

(i) Would you prefer a normal birth with a painless delivery method for your current pregnancy?
  (a) Yes
  (b) No
(ii) Please, mark the most important reason for you in choosing the methods of painless childbirth
  (a) Intolerance to labour pain
  (b) Recommended by doctors
  (c) Other
(iii) Please, mark the most important reason in not choosing the methods of painless childbirth for you.
  (a) Desire for a natural method of childbirth
  (b) The idea that the method will not reduce the labour pain
  (c) Concern that the method can damage the baby and mother
  (d) Other
